# 2024 Recommendations for Validation of Noninvasive Arterial Pulse Wave Velocity Measurement Devices

**DOI:** 10.1161/HYPERTENSIONAHA.123.21618

**Published:** 2023-11-17

**Authors:** Bart Spronck, Dimitrios Terentes-Printzios, Alberto P. Avolio, Pierre Boutouyrie, Andrea Guala, Ana Jerončić, Stéphane Laurent, Eduardo C.D. Barbosa, Johannes Baulmann, Chen-Huan Chen, Julio A. Chirinos, Stella S. Daskalopoulou, Alun D. Hughes, Azra Mahmud, Christopher C. Mayer, Jeong Bae Park, Gary L. Pierce, Aletta E. Schutte, Elaine M. Urbina, Ian B. Wilkinson, Patrick Segers, James E. Sharman, Isabella Tan, Charalambos Vlachopoulos, Thomas Weber, Elisabetta Bianchini, Rosa Maria Bruno

**Affiliations:** Department of Biomedical Engineering, Cardiovascular Research Institute Maastricht (CARIM), Maastricht University, Netherlands (B.S.).; Macquarie Medical School, Faculty of Medicine, Health and Human Sciences, Macquarie University, Sydney, NSW, Australia (B.S., A.P.A., I.T.).; 1st Cardiology Department, Hippokration Hospital, National and Kapodistrian University of Athens, Greece (D.T.-P., C.V.).; Université Paris Cité, Inserm, Paris Cardiovascular Research Center (PARCC), France (P.B., S.L., R.M.B.).; Service de Pharmacologie et Hypertension, Assistance Publique–Hôpitaux de Paris (AP–HP), Hôpital Européen Georges Pompidou, Paris, France (P.B., S.L., R.M.B.).; Vall d’Hebron Institut de Recerca, Barcelona, Spain (A.G.).; Centro de Investigación en Red en Enfermedades Cardiovasculares (CIBER-CV), Instituto de Salud Carlos III, Madrid, Spain (A.G.).; Laboratory of Vascular Aging and Cardiovascular Prevention, Department of Research in Biomedicine and Health, University of Split School of Medicine, Croatia (A.J.).; Feevale University, Santa Casa Hospital, Porto Alegre, Brazil (E.C.D.B).; Praxis Dres. Gille/Baulmann, Rheinbach, Germany (J.B.).; Division of Cardiology, Medical University of Graz, Austria (J.B.).; College of Medicine, National Yang Ming Chiao Tung University, Taipei, Taiwan (C.-H.C.).; Cardiovascular Division, University of Pennsylvania Perelman School of Medicine and Hospital of the University of Pennsylvania, Philadelphia, PA (J.A.C.).; Department of Medicine, Research Institute McGill University Health Centre, McGill University, Montreal, QC, Canada (S.S.D.).; Department of Population Science and Experimental Medicine, Institute of Cardiovascular Science, University College London, United Kingdom (A.D.H.).; Department of Internal Medicine, Pharmacology, and Clinical Research, Shalamar Medical and Dental College, Lahore, Pakistan (A.M.).; AIT Austrian Institute of Technology, Center for Health & Bioresources, Medical Signal Analysis, Vienna (C.C.M.).; JB Lab and Clinic, Department of Precision Medicine and Biostatistics, Wonju College of Medicine, Yonsei University, Seoul, Republic of Korea (J.B.P.).; Department of Health and Human Physiology, University of Iowa, IA (G.L.P.).; School of Population Health, University of New South Wales, Sydney, Australia (A.E.S.).; The George Institute for Global Health, Sydney, NSW, Australia (A.E.S., I.T.).; Cincinnati Children’s Hospital Medical Center, OH (E.M.U.).; University of Cincinnati, OH (E.M.U.).; Experimental Medicine and Therapeutics, University of Cambridge, United Kingdom (I.B.W.).; IBiTech-BioMMedA, Ghent University, Belgium (P.S.).; Menzies Institute for Medical Research, University of Tasmania, Hobart, TAS, Australia (J.E.S.).; Cardiology Department, Klinikum Wels-Grieskirchen, Austria (T.W.).; Institute of Clinical Physiology, Italian National Research Council, Pisa (E.B.).

**Keywords:** aorta, cardiovascular diseases, guideline, methods, pulse wave analysis, vascular stiffness

## Abstract

**BACKGROUND:**

Arterial stiffness, as measured by arterial pulse wave velocity (PWV), is an established biomarker for cardiovascular risk and target-organ damage in individuals with hypertension. With the emergence of new devices for assessing PWV, it has become evident that some of these devices yield results that display significant discrepancies compared with previous devices. This discrepancy underscores the importance of comprehensive validation procedures and the need for international recommendations.

**METHODS:**

A stepwise approach utilizing the modified Delphi technique, with the involvement of key scientific societies dedicated to arterial stiffness research worldwide, was adopted to formulate, through a multidisciplinary vision, a shared approach to the validation of noninvasive arterial PWV measurement devices.

**RESULTS:**

A set of recommendations has been developed, which aim to provide guidance to clinicians, researchers, and device manufacturers regarding the validation of new PWV measurement devices. The intention behind these recommendations is to ensure that the validation process can be conducted in a rigorous and consistent manner and to promote standardization and harmonization among PWV devices, thereby facilitating their widespread adoption in clinical practice.

**CONCLUSIONS:**

It is hoped that these recommendations will encourage both users and developers of PWV measurement devices to critically evaluate and validate their technologies, ultimately leading to improved consistency and comparability of results. This, in turn, will enhance the clinical utility of PWV as a valuable tool for assessing arterial stiffness and informing cardiovascular risk stratification and management in individuals with hypertension.

**NOVELTY AND RELEVANCE**

**What Is New?**

Updated recommendations for the validation of devices measuring pulse wave velocity are provided.

Detailed instructions for different types of devices and their reference standards, study population, and data analyses are specified.

**What Is Relevant?**

Pulse wave velocity is an acknowledged measure of hypertension-related organ damage and is useful for cardiovascular risk stratification in general.

However, its use in clinical practice is limited due to a lack of standardization and homogeneity.

**Clinical/Pathophysiological Implications?**

Application of these recommendations by manufacturers and researchers will facilitate a widespread adoption in clinical practice of pulse wave velocity as a valuable tool for cardiovascular risk stratification in individuals with hypertension and beyond.

Arterial stiffness is established as an independent predictor for mortality and cardiovascular risk.^1,2^ Three consensus documents about the pathophysiology of arterial stiffness and its measurement have been previously developed and endorsed by scientific societies.^3–5^ In 2010, the Association for Research into Arterial Structure and Physiology (ARTERY) formulated the first set of recommendations for the validation of arterial pulse wave velocity (PWV) measurement devices.^6^ These recommendations were widely adopted and used for the validation of many devices. However, still, PWV as a parameter has not seen widespread clinical use. In addition, it has become apparent over the years that some aspects of these initial recommendations should be revised and further refined. The task of revision was adopted by 6 scientific societies in the fields of hypertension and arterial physiology. Collaboration was stimulated through the European Cooperation in Science and Technology (COST) Action VascAgeNet.^7,8^ The result is the present set of recommendations, endorsed by those societies. A summary of the major differences between the previous and present recommendations is provided in Table 1.

**Table 1.**
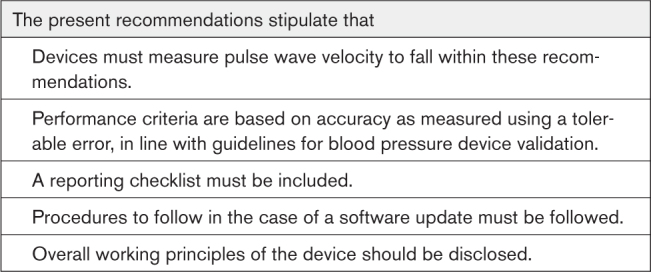
Major Differences Between the Present and 2010 Recommendations^6^

## METHODS

### Data Availability

The authors declare that all supporting data are available within the article and its Supplemental Material. These recommendations were developed using the modified Delphi technique,^9,10^ involving 3 main steps: (1) initial steps, (2) premeeting activities, and (3) a virtual consensus meeting. These steps are detailed in the Methods section in the Supplemental Material.

## SCOPE

The scope of these guidelines (ie, which devices are encompassed) is summarized in Table 2. Further details can be found in the Scope section in the Supplemental Material.

**Table 2.**
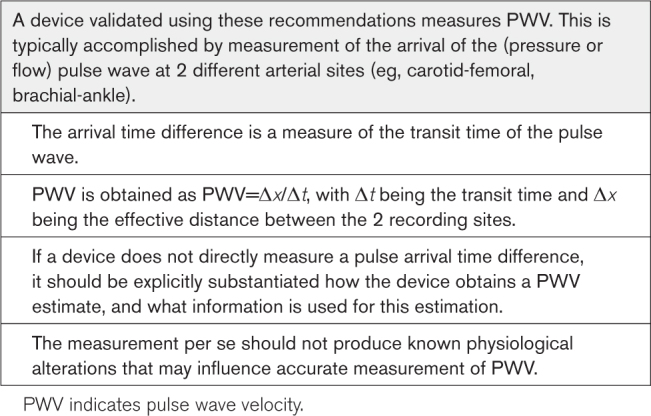
Devices Encompassed by These Recommendations

## REFERENCE STANDARDS

### Choice of Reference Standard

Ideally, only 1 gold standard measurement (eg, invasively recorded PWV) should be used as a reference for the validation of all PWV devices. However, maximum care has to be taken when invasive measurements are performed due to several potential issues.

- Standardization
  - Invasive PWV can be measured synchronously using 2 pressure sensors spaced apart on 1 catheter (preferred) or sequentially using a single catheter (acceptable; pullback method). In the latter case, it is important to ensure that there is minimal variation (<5%) in heart rate or blood pressure (BP) during the sequential recordings (ie, hemodynamic stability), and readings should be made in varying sequence.
  - Catheters can be pressure tip (preferred) or fluid filled (acceptable); fluid-filled catheters require due care with respect to calibration.^11^
  - Due to PWV beat-to-beat variability,^12^ at least 10 heartbeats must be recorded.
  - See the Practical Considerations section for further details.
- Trajectory mismatch: invasive PWV is typically measured from just above the aortic valve to just above the aortic bifurcation. This does not exactly match the carotid-femoral pathway, the most easily accessible pathway for the noninvasive estimation of aortic PWV. More critically, for PWVs involving longer peripheral arterial segments (eg, brachial-ankle PWV [baPWV] or finger-toe PWV), no corresponding invasive PWV can be measured.

In all cases, the reference standard must be relevant to the intended use of the test device. This means that devices claiming to measure aortic PWV should be preferentially validated against invasive aortic PWV. For invasive aortic PWV, low variability between 2 observers was demonstrated,^13,14^ as well as proven prognostic value.^15^ Some technical requirements should be respected.

#### Frequency Content of the Recorded Signal

Detection of the diastolic foot for transit time estimation requires that the information in the pressure waveform is captured accurately and with an excellent signal-to-noise ratio. In particular, recording of at least 20 harmonics of the arterial pressure signal should be attainable,^11^ which, for a maximum heart rate of 180 bpm (3 Hz), amounts to a frequency of the 20th harmonic of 60 Hz. To properly capture this (1) the pressure sensor (in the case of a pressure-tip catheter) or the pressure sensor plus fluid-filled catheter system (in the case of a fluid-filled catheter) should have a sufficiently wide and linear frequency response to pass this frequency and (2) sampling frequency should be at least 120 Hz to adhere to the Nyquist criterion.

#### Foot Detection at High Temporal Resolution

The aforementioned minimum sampling rate of 120 Hz amounts to a sampling interval of 8.3 ms. To minimize quantization errors, foot detection using the intersecting tangent or diastole patching methods should be performed at a subsample time resolution, for example, 1 ms. For further details,^16^ see the Foot Detection at High Temporal Resolution section in the Supplemental Material.

#### Other Considerations

The arterial path length should be determined from the catheters themselves (ie, based on the length of the catheter wire for the pullback method measured in a resolution of millimeters) or, alternatively, from high-quality radiographic images obtained during the procedure.

#### Devices Measuring Carotid-Femoral PWV

For devices whose intended use is to measure carotid-femoral PWV (cfPWV), given the potential issues mentioned above and the complexity of invasive studies, a noninvasive gold standard is also recommended, using as reference devices for which cfPWV has been validated against invasive data and has also provided consistent, robust clinical evidence in large population-based samples of association with future outcomes, such as cardiovascular events and mortality.^1^ Commercial devices that currently fulfill these criteria are, for example, the Complior Analyse device (Alam Medical, Saint Quentin Fallavier, France) and the original Sphygmocor system that used tonometry for both carotid and femoral pulse wave acquisitions (not sold anymore; ATCOR Medical, CardieX Pty, Ltd, Sydney, NSW, Australia). Using as a reference technique to validate devices, Doppler ultrasound or magnetic resonance imaging was not favored by the panel for reasons of data quality and heterogeneity of acquisition protocols. Nevertheless, magnetic resonance imaging along with computed tomography remains the gold standard for path length assessment, ideally using biplanar images for a 3D reconstruction of the centerline. The list of devices that can be used as reference can be subjected to updates in the future if relevant data become available. Table 3 specifies measurement requirements for the reference standards.

**Table 3.**
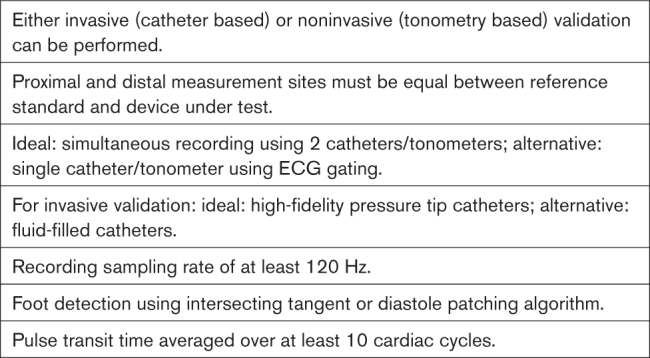
Measurement Requirements for Reference Standards

#### Devices Involving the Use of a Femoral Cuff

Several recent cfPWV devices use a cuff (as opposed to a tonometer) to measure the femoral pressure waveform. In this case, the effective femoral pressure measurement location is more distal than with a tonometer, requiring a correction equation to convert the resulting PWV value to a value that corresponds to cfPWV.^17,18^ Because of the need for such correction, devices using this method should not be used as reference devices for validation of other cfPWV devices but still should be validated according to the protocol proposed in these recommendations. It is advisable that correction equations used are made public and undergo external validation in independent study populations to fully ensure correspondence between the measured values and the true cfPWV. Validation in special populations (ie, children and adolescents) becomes particularly important for this kind of devices.

#### Devices Measuring PWV Along Pathways Other Than Carotid-Femoral

Transit time-based PWV devices measuring different pathways than cfPWV (eg, baPWV) are increasingly used and have demonstrated association with cardiovascular events and significant risk reclassification.^19^ baPWV is systematically higher than cfPWV by 2 to 9 m/s in studies from Asia and the United States.^20^ The magnitude of this difference is clinically relevant (this panel agrees that 1.0 m/s is the minimum clinically important difference for cfPWV^1^; see Sample Size and Selection of Participants section), but the evidence related to these aspects presents considerable heterogeneity. This appears to be caused not only by differences in path length assessment but also by the presence of more muscular arteries in the path for baPWV. Sugawara et al^21^ showed that baPWV recalculated using the magnetic resonance imaging–based path length was 11% lower than that derived from the height-based path length but was still 45% greater than cfPWV. In such circumstances, the same reference standard for devices measuring cfPWV and baPWV could not be supported. We, thus, recommend that baPWV measured with a new device is validated against another device measuring baPWV, which has provided robust clinical evidence, in parallel with what is recommended for validating cfPWV devices. To date, the BP-203RPE device (Omron Healthcare Co, Ltd, Kyoto, Japan) fulfills these criteria.^19^

Notably, some devices measure a PWV over 1 arterial segment and then estimate PWV for another arterial segment. For example, a device may measure baPWV and then estimate cfPWV using a conversion formula. We recommend that measured and estimated parameters are clearly and appropriately labeled by manufacturers and such conversion formulas are made public and undergo external validation in independent study populations.

The previous section specifies the requirements for a reference standard device. A technical validation study should always be performed by comparing a device under test to a reference standard device. This has an important implication; namely, once a device is validated, it does not automatically qualify as a reference standard device. This choice was made to limit the potential of error propagation.

### Validation of Devices Based on Novel Principles and Arterial Segments

As outlined under the Scope section and in Table 2, this document mainly entails the validation of devices that directly measure the transit time. Some devices measure the transit time along an arterial segment for which no reference standard device is available.^22,23^ In this case, although a formal technical validation is not possible, we do recommend using the protocol outlined in these recommendations to make a comparison against an available device supported by clinical prognostic data; this comparison should be clearly reported in the validation document. Importantly, these devices cannot claim to measure cfPWV or aortic PWV; it should be clearly stated that they solely provide an estimation. It is advisable that algorithms and conversion formulas are made public and undergo external validation in independent study populations (in parallel to what was stated earlier for baPWV). Another class of devices typically reports a derived quantity instead of PWV, for example, cardio-ankle vascular index (CAVI).^24^ Validation of these devices is encompassed by these recommendations because this kind of measure complies with the scope of this document. In this case, a validation using the underlying PWV metric (heart-to-ankle PWV for CAVI) is recommended. Since the measured parameter is intrinsically different from cfPWV, it is impossible to infer an equivalent predictive value and a similar clinical use. Specific clinical investigations should be designed to ascertain the intended use of these metrics.

Several devices report a PWV value based on other information than a measured transit time, such as pressure waveform analysis acquired on a single site, combined with mathematical algorithms together with clinical variables, such as age, sex, and BP.^25–27^ Such devices do not provide a measured PWV but estimate it based on different parameters. Furthermore, an increasing number of machine learning algorithms are developed for the estimation of PWV or more in general of vascular age.^28,29^ Indeed, the relationship between measured PWV and its estimate obtained by these methods may vary according to many known and unknown, acute and chronic, and physiological and pathological conditions. The use of the validation protocol described in these recommendations, focused on the assessment of accuracy and precision,^30^ though encouraged for these devices too, does not address all the issues related to their reliability (eg, their ability to track PWV changes and their long-term prognostic value). To avoid confusion, particularly for users new to the field, we encourage device manufacturers to specify clearly that the metric provided is an estimated PWV. Specific guidelines need to be developed for the validation of these kinds of devices or algorithms, in parallel to what has been done, for example, for cuffless BP devices.^31^ Indeed, some of these new technologies fall more appropriately in the category of artificial intelligence–based prediction models because they are meant to accurately stratify individual cardiovascular risk rather than to measure a physical quantity such as PWV. For this category of devices too, additional clinical investigations to establish their clinical value need to be designed.

## SAMPLE SIZE AND SELECTION OF PARTICIPANTS

The sample size for any validation procedure comparing 2 PWV devices should be sufficiently large to estimate the probability of tolerable error reliably. We chose 1 m/s as a tolerable error for PWV measurement, with error defined as the mean difference between readings from device 1 and device 2 over 3 measurements; see the Data Analysis section. The 1-m/s value was chosen by the panel of experts as the minimum clinically important difference. In an individual-participant meta-analysis, a change in PWV of 1 m/s was associated with a hazard ratio for cardiovascular events of 1.07 (95% CI, 1.02–1.12) for men aged 60 years with no risk factors.^1^ The panel of experts also decided that devices for which the error is >1.0 m/s but ≤1.5 m/s will be categorized as having acceptable performance. The choice of this threshold, while being wide, aligns with the findings on the reproducibility of a single device.^32^

In accordance with the standard used for BP-measuring devices,^33^ at least 85% of the sample measurements should be within the tolerable error. The Figure illustrates how the error varies as a function of the mean difference between 2 devices and the SD of this difference. Table S1 presents the acceptable limit for SD of the difference as a function of the mean difference. This probability, however, is subject to error when calculated from a sample, and the sample size should be large enough to determine it precisely. With a sample size of n≥85, one can be 90% confident that the probability of tolerable error does not exceed ±7% margin of error, or, in other words, the true probability of tolerable error is at least 78%. This same level of precision is used in the American National Standards Institute/Association for the Advancement of Medical Instrumentation/International Organization for Standardization standard for BP measurement devices.^34^ To account for dropout and ensure that n=85 individuals without missing data are available for final analyses, we recommend enrolling at least n=90 individuals.

**Figure.**
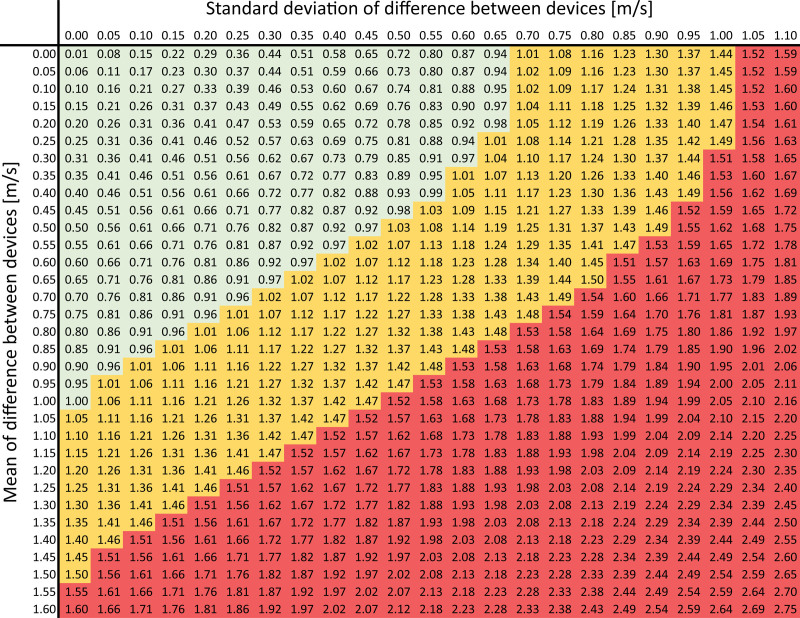
**Measurement error (m/s) for which the probability of observing such error or less is 85% (numbers on colored background).** Colors (device validation outcome): green: good (error ≤1.0 m/s); orange: acceptable (error ≤1.5 m/s); and red: fail (error >1.5 m/s). Before looking up a combination of mean and SD, round up both values to 0.05-m/s precision (eg, 0.41 m/s is rounded to 0.45 m/s; 0.47 m/s is rounded to 0.50 m/s). For example, a mean difference of 0.36 m/s (rounded to 0.40 m/s) and an SD of that difference of 0.44 m/s (rounded to 0.45 m/s) correspond to an error of 0.88 m/s. This implies that, on average, 85% of the validation measurements are within ±0.88 m/s of the reference measurement. Because 0.88≤1.0 m/s, this device passes validation with good accuracy.

A sufficient sample size is also needed to provide a reasonable range of measured values, but it should not be too large to be impractical. A consensus was reached that a validation study should include only participants aged ≥18 years. A further consideration is having a sufficient range of reference PWV readings to detect any tendency for the difference between devices to depend on the mean value. In case a cfPWV device is validated, the population shall have ≥5% of the reference PWV readings ≤6 m/s, ≥5% with ≥10 m/s, and ≥20% with ≥8 m/s. When a device measures or estimates PWV for another arterial bed, these cutoffs should be scaled accordingly. Since age is the predominant determinant of PWV, we suggest a relatively even spread among the following 4 age groups (with ≥10 individuals in any one age group): <30, 30–49, 50–69, and ≥70 years. Based on current evidence, sex seems to have little impact on PWV,^35^ but this may not be true for future techniques. Thus, we recommend ≥40% men and ≥40% women in each of the aforementioned age groups. Although heart rate may influence PWV, we do not recommend any specific ranges for heart rate because its effect seems to be mediated largely by the effect on BP.^36^

Participants receiving vasoactive medications should not be excluded a priori. However, validation measurements should not be recorded within the time window when there may still be effects from the acute administration of vasoactive medications.^4^ Participants should be in sinus rhythm and not be pacemaker dependent at the time of measurement as beat-to-beat cardiac contraction variation, intermittent uncontrolled HR, and change in mean BP in atrial fibrillation could influence the reliable assessment of PWV.^37^ Similarly, individuals with a body mass index ≥40 kg/m^2^ are to be excluded from the regular validation population due to problems with accurate path length measurement,^38^ as well as those in whom palpation of the relevant arteries is impossible. Furthermore, patients with clinically relevant arterial stenosis between the 2 sites of measurement or severe aortic valve stenosis are to be excluded due to the possibility of increased transit time because of the severe obstruction.^39^

A list of the subject selection criteria is presented in Table 4.

**Table 4.**
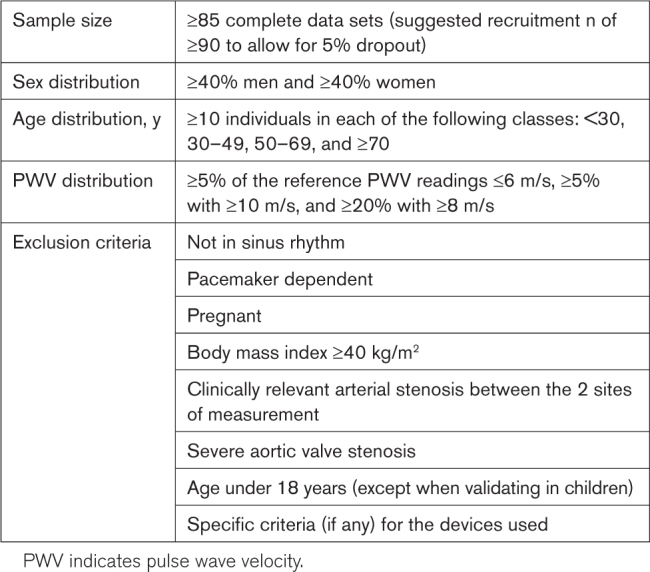
Subject Selection Criteria for Validation Studies

### Special Populations

The present protocol is applicable also in special populations, which we defined as populations in which there is theoretical and clinical evidence of different accuracy of PWV devices. These include, for example, pregnant/preeclamptic women,^40^ obese participants, and children/adolescents.^41^ Further details can be found in the Sample Size and Selection of Participants in Special Populations section in the Supplemental Material.

## PRACTICAL CONSIDERATIONS

### General

- The investigators performing the validation study should be fully trained in the use of both the reference device and the device under test.
- The testing environment should be quiet and provide enough privacy for the participants to feel comfortable. Participants should be made familiar with the PWV measurement equipment to be used. It is suggested that at least 1 dummy recording is performed before the actual validation protocol is started.
- When sequential recordings referenced to the ECG are used, it is important to ensure that there is minimal variation (<5%) in heart rate or BP during the sequential recordings (ie, hemodynamic stability), and readings should be made in varying sequence.
- A minimum recording time of 10 cardiac cycles for each measurement site should be used.
- Transit time should be determined from the waveforms using an intersecting tangent algorithm or diastole patching algorithm, as these have been shown to be more accurate and less influenced by changes in wave shape or wave reflection than other (eg, second derivative based) methods.^42,43^
- PWV values should be recorded in a precision of at least tenths of meters per second (ie, 1 decimal when values are recorded in meter per second).

### Carotid-Femoral PWV

- Preference is given to recordings from the right carotid and right femoral arteries.
- The carotid-femoral path length estimate used in calculating the reference PWV should match the estimate that is used in the device under test. Commonly used estimates include the subtraction method (subtracting the sternal notch-to-carotid distance from the sternal notch-to-femoral distance) and the 80%-of-direct-distance method (multiplying the direct carotid-to-femoral distance by 80%). Reproducibility of distance measurements should be reported too because it represents an important source of variability of PWV measurements.

## CONFLICT OF INTEREST AND TRANSPARENCY PROMOTION

To avoid industry bias, the validation study should ideally be conducted by investigators who are independent of the manufacturers of the device being tested. In general, any possible interest (eg, grants or contracts, consulting fees, stocks, speaker honoraria, patents, or other commercial interests) must be declared. To improve the transparency and openness of science, we also recommend that the original data of validation studies are made available in a public repository. This also aligns with the emerging approach of Medical Devices Regulation frameworks, such as the publicly available EUDAMED database under Medical Devices Regulation (EU) 2017/745. Independent replication studies are also encouraged to improve reproducibility of results.

## PROCEDURE

The procedure for PWV device validation is detailed in Table 5. It is important to describe in advance quality criteria to consider a measurement as failed and report the number of measurements discarded from the analysis and for which reason.

**Table 5.**
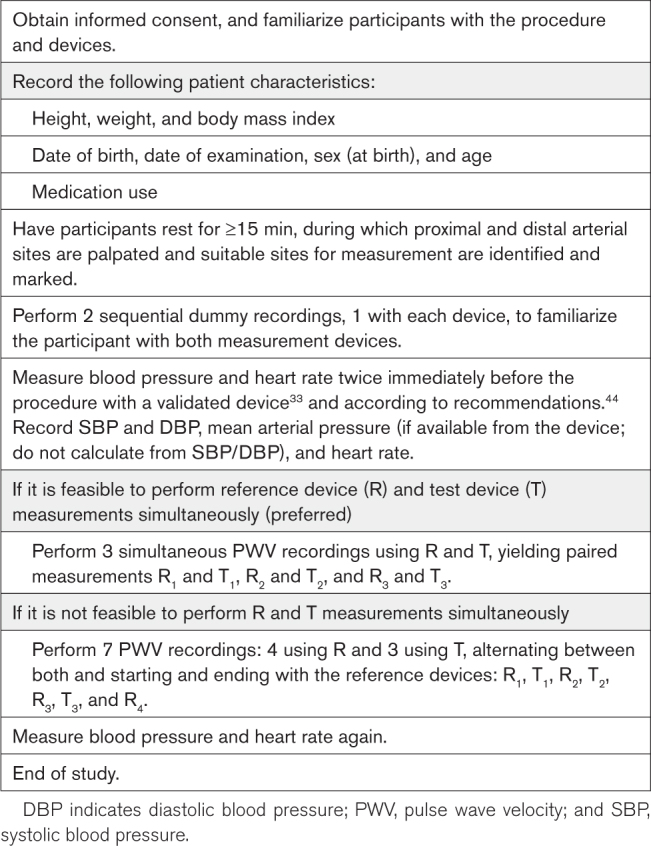
Validation Protocol

## DATA ANALYSIS

Data analysis and the associated calculations^45^ are described in the Supplemental Material. Briefly, the comparison of a device under test with a reference device is made as follows. If a simultaneous PWV measurement using the device under test (T) and the reference device (R) is feasible, T and R measurements will be performed simultaneously, yielding 3 pairs of T and R measurements (R_1_ and T_1_, R_2_ and T_2_, and R_3_ and T_3_; Table 5). If simultaneous measurement is not feasible, for each measurement of the device under test (T_1_, T_2_, and T_3_), the average of the 2 reference measurements closest in time (R_1_, R_2_, R_3_, and R_4_) is used as a comparator (Table 5), in parallel to what is recommended for validation of BP monitors.^33,34^ For a device to pass comparison to a reference device for a given measured mean difference in PWV, the SD should obey the respective cutoff value in Table S1, ensuring at least an 85% probability of a tolerable error of 1.0 m/s (good accuracy) or 1.5 m/s (acceptable accuracy), respectively (Figure).

## REPORTING

### General

The overall principles behind the PWV measurement should be disclosed and illustrated. This includes, for example, a description of the fiducial point used on the waveform, the method used to detect this fiducial point, and whether/which additional information is used to compute the PWV. If proprietary aspects play a role, this should be stated, and when applicable, references to patents should be given. If algorithms used are trained based on experimental data sets (eg, neural networks, statistical regression models), it is suggested for such data sets to be disclosed. When the reported PWV is (partly) dependent on training or model fitting (eg, regression), the participants whose data were used for training should not be included in the validation study (ie, training and validation sets should be separate). A completed reporting checklist (Table S2) should accompany each device validation study publication. The validation study results should be made publicly available by publication in the peer-reviewed literature. We strongly suggest that the validation study is openly available to maximize accessibility. Raw data should be deposited in a publicly accessible repository. Mean and SD of BP and heart rate, both before and after the procedure, should also be reported.

### Comparison With Reference Device

A Bland-Altman plot^46^ (where the differences between the 2 techniques are plotted against the averages of the 2 techniques) should be presented, including the mean difference (horizontal line and numerical value), SD of the difference (numerical value), and mean−2 SD to mean+2 SD interval (horizontal dashed lines). We suggest also showing a regression line (and associated equation, *R*^ 2^, and *P* value) in the Bland-Altman plot, facilitating the assessment of a possible relationship between the mean difference and the (average) distance. Notably, a scatterplot of the raw data and the associated regression line, *R*^ 2^, and *P* value can be shown, but it should not be used to infer agreement between the device under test and the reference device.^47^ The analyses described here should be performed (1) on the full sample of participants, (2) stratified by sex, and (3) stratified by age category (<30, 30–49, 50–69, and ≥70 years). An analysis stratified by the use of vasoactive drugs is also advisable.

### Test-Retest Reproducibility

Precision can be determined as the reproducibility of the obtained data by a single device. In addition, for this end point, the maximum allowed difference between repeated measurements is a crucial point; the variation between measurements repeated with the same device (on the same participant and in similar conditions) should be lower than the minimum clinically important difference. A Bland-Altman analysis with a predefined clinical agreement limit is recommended also for this validation.^46^ It is worth noting that negligible bias is expected for data obtained by the same device by the same observer. The precision of repeated measurements should also be reported in terms of coefficient of variation (SD of 2 measurements divided by their average).^48^

## DEVICE SOFTWARE VERSION CONTROL

Studies have shown that changes in the software and hardware used in PWV devices may significantly alter their output.^42,49^ Hence, it is important that, in a validation study, a consistent software and hardware version of the device under test is used and reported. Recommendations for handling device software changes are given in the Device Software Version Control section in the Supplemental Material.

## CONCLUSIONS

The present document serves as an updated guide for the validation of noninvasive arterial PWV measurement devices. It is hoped that this document will encourage both users and developers of PWV measurement devices to critically evaluate and validate their technologies, ultimately leading to improved consistency and comparability of results.

### Perspectives

Broad acceptance and implementation of these recommendations will facilitate broader adoption of PWV systems within clinical practice and the market, including reimbursement from public health care systems and health insurance. This document will be periodically updated to include novel measurement modalities available in the future.

## ARTICLE INFORMATION

### Sources of Funding

B. Spronck received funding from the European Union’s Horizon 2020 Research and Innovation Program (No. 793805). A. Guala received funding from the La Caixa Foundation (LCF/BQ/PR22/11920008). A. Jerončić was funded by the Croatian Science Foundation (No. HRZZ IP-2018-01-4729). J.A. Chirinos was supported by National Institutes of Health grants R01-HL121510, U01-TR003734, 3U01-TR003734-01W1, U01-HL160277, R33-HL146390, R01-HL153646, K24-AG070459, R01-AG058969, R01-HL104106, P01-HL094307, R03-HL146874, R56-HL136730, R01-HL155599, R01-HL157264, and 1R01-HL153646-01. S.S. Daskalopoulou is a senior clinician-scientist supported by a Fonds de recherche du Québec-Santé Senior Salary award. VascAgeNet (CA18216: Network for Research in Vascular Ageing) is supported by European Cooperation in Science and Technology (COST; www.cost.eu). G.L. Pierce was funded by grants from the National Institutes of Health (R01-AG063790) and the American Heart Association (19TPA34910016). A.E. Schutte was supported by a National Health and Medical Research Council Leadership Investigator Grant (application ID: 2017504). I. Tan was cofunded by ATCOR Medical.

### Disclosures

A.P. Avolio is a member of the Scientific Advisory Board of CardieX. P. Boutouyrie has participated in the past in the validation of multiple devices, among them Popmètre and WITHINGS Body Cardio. P. Boutouyrie has research grants with WITHINGS for the development of connected health devices. P. Boutouyrie, P. Segers, and R.M. Bruno are involved as researchers in an H2020 project working on the development of an integrated silicon photonics-based device that aims to measure pulse wave velocity. S. Laurent received research grants and honoraria as a speaker or chairperson and has been on the advisory board of Servier Laboratories, SOM Biotech, and WITHINGS. J. Baulmann has an interest in Redwave Medical GmbH and received equipment and lecture fees from IEM GmbH, BPLab, SMT Medical GmbH & Co, SOT Medical Systems, and Tensiomed. J.A. Chirinos received University of Pennsylvania research grants from the National Institutes of Health, Fukuda-Denshi, Bristol-Myers Squibb, Microsoft, and Abbott. J.A. Chirinos was named inventor in a University of Pennsylvania patent for the use of inorganic nitrates/nitrites for the treatment of heart failure and preserved ejection fraction and patent applications for the use of biomarkers in heart failure. J.A. Chirinos received payments for editorial roles from the American Heart Association, the American College of Cardiology, and Wiley. J.A. Chirinos received research device loans from ATCOR Medical, Fukuda-Denshi, Unex, Uscom, NDD Medical Technologies, Microsoft, and MicroVision Medical. S.S. Daskalopoulou is a cofounder of the start-up PLAKK that aims to identify individual atherosclerotic plaque instability and stroke risk using artificial intelligence methods. C.C. Mayer is an inventor (not holder) of a patent that is partly used in the ARCSolver algorithm in the Mobil-O-Graph 24h PWA Monitor (IEM GmbH). A.E. Schutte received speaker honoraria from Servier, Novartis, Abbott, Sanofi, Omron, Aktiia, and Medtronic. E.M. Urbina was on the Data Safety Monitoring Board of Astellas, Inc, and received an honorarium from Targus Medical, Inc. I. Tan was an employee of ATCOR Medical. E. Bianchini is a cofounder of QUIPU s.r.l., a spin-off company of the Italian National Research Council and the University of Pisa developing software for medical devices.

### Supplemental Materials

Supplemental Materials

Tables S1 and S2

Figure S1

## Supplementary Material


