# Supplementary material for: 2024 Recommendations for Validation of Noninvasive Arterial Pulse Wave Velocity Measurement Devices
Source: Hypertension. 2023 Nov 17;81(1):183–92. doi: 10.1161/HYPERTENSIONAHA.123.21618 (PMC10734786; doi:10.1161/HYPERTENSIONAHA.123.21618)
Supplement: Supplementary file 1 [file hyp-81-183-s001.pdf]

2024 Recommendations for Validation of Noninvasive Arterial Pulse Wave Velocity Measurement Devices

Supplemental Material

Bart Spronck
Dimitrios Terentes-Printzios
Alberto P. Avolio
Pierre Boutouyrie
Andrea Guala
Ana Jerončić
Stéphane Laurent
Eduardo C.D. Barbosa
Johannes Baulmann
Chen-Huan Chen
Julio A. Chirinos
Stella S. Daskalopoulou
Alun D. Hughes
Azra Mahmud
Christopher C. Mayer
Jeong Bae Park
Gary L. Pierce
Aletta E. Schutte
Elaine M. Urbina
Ian B. Wilkinson
Patrick Segers
James E. Sharman
Isabella Tan
Charalambos Vlachopoulos
Thomas Weber
Elisabetta Bianchini
Rosa Maria Bruno

On behalf of
The Association for Research into Arterial Structure and Physiology (ARTERY)
The European Society of Hypertension Working Group on Large Arteries
European Cooperation in Science and Technology (COST) Action VascAgeNet
North American Artery Society
ARTERY LATAM
Pulse of Asia
Society for Arterial Stiffness — Germany-Austria-Switzerland (DeGAG)

Addresses for correspondence

Bart Spronck
Dept. of Biomedical Engineering
Cardiovascular Research Institute Maastricht (CARIM)
Maastricht University
P.O. Box 616, 6200 MD, Maastricht
The Netherlands
b.spronck@maastrichtuniversity.nl

Rosa Maria Bruno
INSERM U970 Team 7
Paris Cardiovascular Research Centre
Université de Paris AP-HP, Pharmacology Unit
Hôpital Européen Georges Pompidou
56 Rue Leblanc, 75015, Paris
France
rosa-maria.bruno@inserm.fr

METHODS

These recommendations were developed using the Modified Delphi technique,[9,10] involving 3 main steps: (1) initial steps, (2) premeeting activities, and (3) a virtual consensus meeting. These steps are detailed in the following paragraph.

1. Initial steps: after having established the need for an update of existing recommendations (see the Introduction section), a literature review was performed by selected experts. This phase culminated in a session organized at the 2021 European Society of Hypertension (ESH) meeting (April 2021). The methodology and literature recommended by the speakers was crucial to establish the following steps.
2. Premeeting activities: an executive group (B.S., R.M.B., D.T.-P., and E.B.) was established, acting as facilitator in the conduction of the Delphi process. This committee selected panelists based on their expertise in the field and as representatives of scientific societies with a specific interest in pulse wave velocity (PWV). Inclusiveness criteria in terms of gender and representation of different geographic areas were also used. The executive group developed a questionnaire to be submitted to the panelists. The questionnaire included 7 sections (Technology, Reference Standards, Validation Cohorts, Practical Considerations, Data Analysis, Clinical Validation, and Other Issues) with 2–6 items each. Based on the responses, the executive group developed a first synthesis of the responses, identifying elements of consensus and disagreement, which was resubmitted to the panelists for further discussion. Questionnaire development and synthesis were performed during several videoconferences and email exchanges within the executive group.
3. Consensus meeting: a consensus meeting took place virtually in May 2022. The most critical issues were discussed collectively. Final decisions were taken by majority, also by employing anonymous polls.

SCOPE

The present recommendations focus mainly on devices measuring PWV through the measurement of a transit time. For these recommendations to be applicable, the overall principles behind the PWV measurement must be known and reported (**Table 2**). In addition, the measurement per se should not produce known physiological alterations that may influence accurate measurement of PWV, eg, carotid tonometry should be performed with minimal applanation force to prevent spurious baroreceptor activation, and cuffs should not occlude blood flow. PWVs can be measured along several arterial segments, of which the carotid-femoral and brachial-ankle are the most commonly used. In principle, these recommendations encompass all arterial segments where a PWV can be measured.

For the validation of devices, performance in terms of precision (ie, repeatability/reproducibility) and accuracy (ie, theoretically closeness to real value, practically agreement with reference) are taken into consideration because these features determine the reliability and validity of a device in clinical practice.[30] Validation is a fundamental prerequisite for a device to be clinically useful. For this reason, structured standardized protocols providing

evidence of the performance of a system need to be implemented starting from the first phase of its design, including research prototypes, to the final marketed medical device.

Clear and replicable protocols, implementing a robust technical validation process agreed by the main representatives of the concerned scientific community could allow a broader adoption of PWV devices in clinical practice. As for blood pressure (BP) measuring devices,[33] a future initiative involving international organizations delivering standards for the stakeholders acting in the field is desirable and could speed up introduction and application of these recommendations. Testing whether a technically validated device predicts relevant clinical outcomes in terms of diagnostic accuracy or therapeutic efficacy is beyond the scope of these recommendations.

FOOT DETECTION AT HIGH TEMPORAL RESOLUTION

The minimum sampling rate of 120 Hz stated in these recommendations amounts to a sampling interval of 8.3 ms. To minimize quantization errors, foot detection using the intersecting tangent or diastole patching methods should be performed at a subsample time resolution, eg, 1 ms. This is possible since foot detection is not directly limited by the sampling interval if the sampling rate is sufficient to capture the signal information. This can be understood from the fact that the detection of the foot is determined by more than just 1 sample. For example, for the intersecting tangent method, the maximum-slope point typically will be somewhere between 2 recorded samples. By using a smooth (eg, splines) interpolation function to upsample the recorded signal, one can determine this point at subsample resolution and reduce quantization error. This similarly holds for the diastolic foot. Taken together, this leads to an effective time resolution of foot detection that is better than the sampling resolution (8.3 ms in this case). This approach is similar to subsample delay estimation methods, which are used widely in other fields, eg, in delay estimation from ultrasound echo data.[16]

SAMPLE SIZE AND SELECTION OF PARTICIPANTS IN SPECIAL POPULATIONS

General Population and Special Population Studies

The present protocol is applicable also in special populations, which we defined as populations in which there is theoretical and clinical evidence of different accuracy of PWV devices.

Special population studies with smaller sample sizes should be performed only after a full general population study has been successfully completed. If the device is intended only for a special population (and, hence, has not been validated in the general population), a full 85-participant study is required. Special population study data should be analyzed and reported independently of the general population study data. Device performance criteria for special populations are the same as for the general population.

There is no agreed procedure for PWV validation in participants with atrial fibrillation, which at present constitutes an exclusion criterion for PWV measurement because of low accuracy.[37] Pregnant/preeclamptic women are considered as a special population of interest due to the

complexity of assessment of PWV in this population based on the constantly changing underlying hemodynamic conditions (ie, trimester, preeclampsia).[40] Participants with severe obesity (body mass index ≥40 kg/m^2) are also considered as a special population of interest. As far as obesity is concerned, inaccuracy of the measurement by tape measure is attributed to inaccurate distance measurement.[38] We thus recommend that technical validation be performed on both transit time and PWV in obese patients.

Sample Size for Special Population Studies

According to the aforementioned sample size calculation, a sample of 90 enrolled (85 complete) participants is desirable for each special population. If a device was validated in the general population, an additional special population validation study can be performed in ≥35 special population participants. The PWV distribution criterion for general population studies as given above cannot be directly applied in most special populations. Instead, tailored criteria need to be defined for each special population because of their different representative PWV levels.

Devices Intended for Use in Both Adults and Children

For devices intended for the general population as well as for children/adolescents, complete measurements in 35 participants aged 3–12 years should be included, together with complete measurements in 50 participants aged ≥13 years.[33] To ensure a broad range in PWV values, we suggest striving for the following BP ranges.[41] If the child is ≥13 years of age, there should be ≥5% with systolic BP (SBP) ≤100 mmHg, ≥5% with SBP ≥130 mmHg, and ≥20% with SBP ≥120 mmHg. For children ≤12 years of age, percentiles should be used. Therefore, the criteria for BP distribution are 5% with SBP ≤50th percentile for age, sex, and height, 5% with SBP ≥95th percentile, and 20% with SBP ≥90th percentile. Further to the formal analysis of the total 85-participant sample, the mean PWV difference (test versus reference device) and its SD shall in addition be reported separately for subgroups aged ≤12 and ≥13 years.

<u>DATA ANALYSIS</u>

Comparison With Reference Device

If a simultaneous PWV measurement using the device under test (T) and the reference device (R) is feasible, T and R measurements are performed simultaneously, yielding 3 pairs of T and R measurements (R_1-and-T_1, R_2-and-T_2, R_3-and-T_3; **Table 5**). The mean difference for individual subject *i* over repeated measurements $j = 1, 2, 3$ is then calculated as

$$x_i = \frac{1}{3}\sum_{j=1}^{3}\left(\mathrm{PWV}_{i,\mathrm{T}_j} - \mathrm{PWV}_{i,\mathrm{R}_j}\right).$$

If simultaneous measurement is *not* feasible, for each measurement of the device under test (T_1, T_2, and T_3), the average of the 2 reference measurements closest in time (R_1, R_2, R_3, and R_4) is used as a comparator (**Table 5**), in parallel to what is recommended for validation of BP monitors:[33,34]

- T_1 is compared to $(R_1+R_2)/2$

- T_2 is compared to $(R_2+R_3)/2$
- T_3 is compared to $(R_3+R_4)/2$

Hence, the mean difference for individual subject *i* over repeated measurements $j = 1, 2, 3$ is calculated as

$$x_i = \frac{1}{3}\sum_{j=1}^{3}\left(\mathrm{PWV}_{i,\mathrm{T}_j} - \frac{\mathrm{PWV}_{i,\mathrm{R}_j}+\mathrm{PWV}_{i,\mathrm{R}_{j+1}}}{2}\right).$$

Once x_i has been calculated, the mean difference over all subjects is given by

$$\mathrm{mean}_{\mathrm{diff}} = \frac{1}{n}\sum_{i=1}^{n} x_i \ ,$$

with n the number of subjects included in the study. The SD of these differences is given by

$$\mathrm{SD}_{\mathrm{diff}} = \sqrt{\frac{1}{n-1}\sum_{i=1}^{n}(x_i - \mathrm{mean}_{\mathrm{diff}})^2} \ .$$

For a device to pass comparison to reference device for a given measured mean difference in PWV, the SD should obey the respective cut-off value in **Table S1**, ensuring at least an 85% probability of a tolerable error of 1.0 m/s (good accuracy) or 1.5 m/s (acceptable accuracy), respectively (also see **Figure 1**).

Test-Retest reproducibility

Intra-observer test-retest reproducibility is quantified using the within-subject SD and the corresponding coefficient of variation.[45] First, SDs within the individual subjects are calculated:

$$\mathrm{SDrep}_i = \sqrt{\frac{1}{m-1}\sum_{j=1}^{m}\left(\mathrm{PWV}_{i,\mathrm{T}_j} - \mathrm{mean}_i\right)^2} \ ,$$

with m the number of repetitions per subject (typically 3), and mean_i subject i's mean, defined as

$$\mathrm{mean}_i = \frac{1}{m}\sum_{j=1}^{m}\mathrm{PWV}_{i,\mathrm{T}_j} \ .$$

Together, this yields the overall within-subject SD as

$$\mathrm{SD}_{\mathrm{within}} = \sqrt{\frac{\sum_{i=1}^{n}\mathrm{SDrep}_i^{\,2}(m_i-1)}{\sum_{i=1}^{n}(m_i-1)}} \ ,$$

with m_i the number of repeated measurements for subject i. When 3 measurements are available for each subject ($m_i = 3$ for all i), these equations simplify to

$$\mathrm{SDrep}_i = \sqrt{\frac{1}{2}\sum_{j=1}^{3}\left(\mathrm{PWV}_{i,T_j} - \mathrm{mean}_i\right)^2} \ ,$$

$$\mathrm{mean}_i = \frac{\mathrm{PWV}_{i,\mathrm{T}_1}+\mathrm{PWV}_{i,\mathrm{T}_2}+\mathrm{PWV}_{i,\mathrm{T}_3}}{3}, \text{ and}$$

$$\mathrm{SD}_{\mathrm{within}} = \sqrt{\frac{\sum_{i=1}^{n}\mathrm{SDrep}_i^{\,2}}{n}} \ .$$

A coefficient of variation is subsequently calculated using

$$\mathrm{CV}_{\mathrm{within}} = \frac{\mathrm{SD}_{\mathrm{within}}}{\mathrm{mean}}, \text{ with}$$

$$\mathrm{mean} = \frac{1}{n}\sum_{i=1}^{n}\mathrm{mean}_i \ .$$

Although intra-subject test-retest reproducibility should be calculated and reported, in line with the current BP device validation guidelines,[33,34] no formal cut-offs are defined for this reproducibility.

DEVICE SOFTWARE VERSION CONTROL

Studies have shown that changes in the software and hardware used in PWV devices may significantly alter their output.[42,49] Hence, it is important that in a validation study, a consistent software and hardware version of the device under test is used and reported. If a software update includes substantial changes that might impact results (eg, change in software architecture, algorithm, etc.), this implies that a device validated using an earlier software version should be revalidated using the new version (**Figure S1**). If possible, the updated device should be revalidated using the measurement data available from the previous validation study. If this is impossible, preferably, revalidation should be performed in the same study population. A complete list of device software versions (‘changelog’) should be kept on the device manufacturer’s website.

SUPPLEMENTAL TABLES

Table S1. Upper limit on the sample standard deviation (SD) to yield at least 85% probability of a tolerable error, calculated for different values of the mean error.

**Mean of difference [m/s]**	**SD of difference must be below... [m/s]**	
	For *good* accuracy	For *acceptable* accuracy
0.0	0.694	1.042
0.1	0.687	1.037
0.2	0.664	1.022
0.3	0.624	0.997
0.4	0.563	0.960
0.5	0.480	0.910
0.6	0.385	0.845
0.7	0.289	0.765
0.8	0.192	0.674
0.9	0.096	0.578
1.0	N/A	0.482
1.1	N/A	0.385
1.2	N/A	0.289
1.3	N/A	0.192
1.4	N/A	0.096
1.5	N/A	N/A
1.6	N/A	N/A

For a device to pass validation, it should show an 85% probability for measurements to have an error ≤1.0 m/s (good) or ≤1.5 m/s (acceptable). For different values of the mean difference, this is accomplished if the SD of the difference is below the abovementioned cut-offs. Before looking up a mean of the difference, round up the mean to 0.1 m/s precision (eg, a value of 0.41 m/s is rounded to 0.5 m/s). Example: a device with a mean difference of 0.70 m/s and an SD of the differences of 0.10 m/s will pass as good (85% tolerable error 0.81 m/s), whereas a device with that same mean difference of 0.70 m/s and a SD of the differences of 0.50 will pass as acceptable (85% tolerable error 1.22 m/s).

Table S2. Reporting checklist for device validation study

✓	**Item**	**Section/ page #**
	Identification of 2 measurement sites used to measure the pressure pulse	
	Method to obtain transit distance	
	Does a reference standard device exist?	
	If “no” to previous item: mention that this is a “preliminary validation study”	
	Which device is used as a reference?	
	Does the device cause any relevant physiological alteration (eg, occlusion of vessel, baroreceptor activation due to carotid artery applanation, etc.)? If so, please specify.	
	Type of signal (velocity, pressure, flow, other)	
	Sampling rate (temporal resolution) of acquired and processed signals	
	Method for detection of fiducial point	
	Additional input information that is used by the device/algorithm for calculating PWV	
	Are anthropometric data used in the estimation process?	
	Are blood pressure data used in the estimation process?	
	Are the algorithms used trained using datasets? If yes, are datasets disclosed?	
	If training data is used: Is the training dataset completely independent of the validation dataset?	
	Have all version numbers of hardware and software used in the validation study been reported, both for the reference device and for the device under test?	
	Have raw PWV data been recorded in a precision of at least tenths of meters per second (ie, 1 decimal when values are recorded in m/s)?	
	Statement that the device was validated using the 2024 version of the validation recommendations for PWV devices	
	Have all funding and conflicts of interest been declared?	
	Has data been uploaded to a publicly accessible repository?	
	Has a study population characteristics table (number of individuals, age/sex/blood pressure distribution) been included?	
	Have Bland-Altman and scatter plots been included comparing the device under test to a reference device?	

SUPPLEMENTAL FIGURES

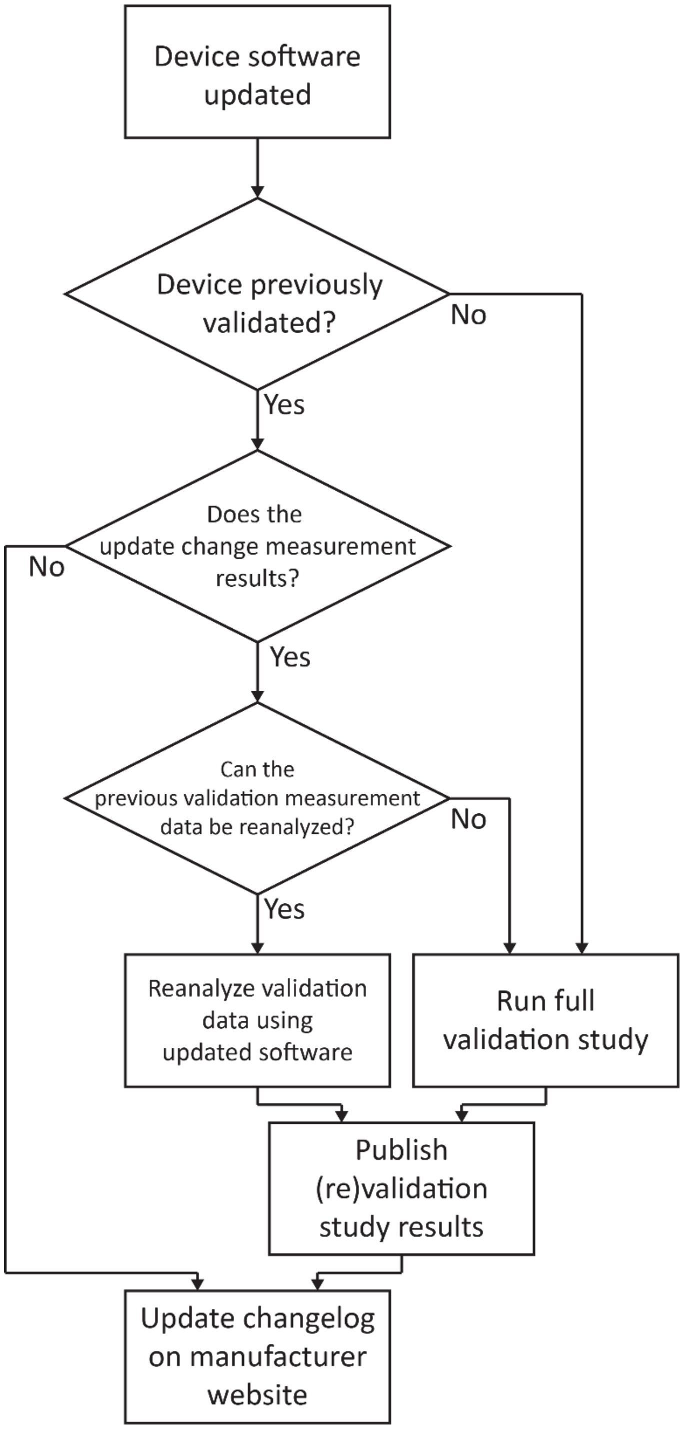

Figure S1. Flowchart to follow at the occurrence of a pulse wave velocity measurement device software version change.
